# Current causes of sudden death in large populations: differences between resuscitated patients and autopsied cases

**DOI:** 10.1093/ehjopen/oeaf125

**Published:** 2025-10-06

**Authors:** Nathan Marimpouy, Céline Guilbeau-Frugier, Anthony Ramirez, Maxime Beneyto, Clement Delmas, Caroline Biendel, Miloud Cherbi, Deborah Foltran, Pierre Mondoly, Jean Timnou Bekouti, Jean Ferrières, Norbert Telmon, Vanina Bongard, Hubert Delasnerie, Anne Rollin, Philippe Maury

**Affiliations:** Department of Cardiology, University Hospital Rangueil, 31059 Toulouse Cedex 09, Toulouse 31059, France; Department of Médecine Légale, University Hospital Purpan, 31300 Toulouse, France; Department of Médecine Légale, University Hospital Purpan, 31300 Toulouse, France; Department of Cardiology, University Hospital Rangueil, 31059 Toulouse Cedex 09, Toulouse 31059, France; I2MC, INSERM UMR 1297, 31059 Toulouse, France; Department of Cardiology, University Hospital Rangueil, 31059 Toulouse Cedex 09, Toulouse 31059, France; Department of Cardiology, University Hospital Rangueil, 31059 Toulouse Cedex 09, Toulouse 31059, France; Department of Cardiology, University Hospital Rangueil, 31059 Toulouse Cedex 09, Toulouse 31059, France; Department of Cardiology, University Hospital Rangueil, 31059 Toulouse Cedex 09, Toulouse 31059, France; Department of Cardiology, University Hospital Rangueil, 31059 Toulouse Cedex 09, Toulouse 31059, France; Department of Cardiology, University Hospital Rangueil, 31059 Toulouse Cedex 09, Toulouse 31059, France; Department of Cardiology, University Hospital Rangueil, 31059 Toulouse Cedex 09, Toulouse 31059, France; Department of Epidemiology, University Hospital Rangueil, 31059 Toulouse, France; Department of Médecine Légale, University Hospital Purpan, 31300 Toulouse, France; Department of Epidemiology, University Hospital Rangueil, 31059 Toulouse, France; Department of Cardiology, University Hospital Rangueil, 31059 Toulouse Cedex 09, Toulouse 31059, France; Department of Cardiology, University Hospital Rangueil, 31059 Toulouse Cedex 09, Toulouse 31059, France; Department of Cardiology, University Hospital Rangueil, 31059 Toulouse Cedex 09, Toulouse 31059, France; I2MC, INSERM UMR 1297, 31059 Toulouse, France

**Keywords:** Sudden cardiac death, Autopsy, Ventricular arrhythmias, Cardiac arrest

## Abstract

**Aims:**

Aetiologies of sudden death (SD) have been reported in autopsied case series and less frequently in resuscitated patients, but large series are scarce and if causes are similar between deceased and surviving patients is unknown.

**Methods and results:**

All successive adult patients with resuscitated SD (*n* = 283) and autopsied SD cases (*n* = 1258) over the last 10 years at our centre were included. Causes were detailed and compared between resuscitated and autopsied cases. Coronary artery disease was present in 87% of resuscitated patients and in 48% of autopsied subjects (*P* < 0.0001). In coronary artery disease patients, an acute coronary event was present in 85% of resuscitated patients vs. 22% of autopsied cases (*P* < 0.0001).

No coronary artery disease was present in 13% of resuscitated patients (42% cardiomyopathy, 58% primary electrical disease) and noncardiac causes were absent. In autopsied cases, some cardiomyopathy was present in 19%, noncardiac causes were noted in 16% (pulmonary embolisms, aortic dissections/aortic aneurysm ruptures or strokes, and brain/meningeal haemorrhages) and no apparent cardiac or noncardiac cause for explaining SD was present in 15% (sudden arrhythmic death syndrome).

**Conclusion:**

In this large series of resuscitated and autopsied SD cases, coronary artery disease remains the main aetiology but was significantly less frequent in autopsied cases, with a majority of acute coronary events in resuscitated patients vs. a majority of remote myocardial infarction without fresh thrombus in autopsied cases. Noncardiac causes were present in 15% of autopsies but never in surviving patients.

## Introduction

Sudden death (SD) is defined by an unexpected natural death occurring within <1 h from the onset of symptoms (or within 24 h of last being seen alive when unwitnessed) in a subject without any prior condition that would appear fatal at short term.^1^

SD is mainly related to cardiac causes—‘sudden cardiac death’ (SCD)—after excluding less common vascular, neurological, or respiratory causes and is usually the final result of malignant ventricular arrhythmias.

Although aetiologies of SD have been previously reported in a wide number of autopsied case series, large series are scarce and ancient, while causes of SD may have changed over the years due to evolutive epidemiology and changing cardiac diseases. Furthermore, causes of SD in resuscitated patients remain grossly evaluated, and differences in aetiologies of SD between deceased and resuscitated patients do not seem to have been deeply investigated. A difference in aetiologies between deceased and resuscitated patients may have potential mechanistic and clinical implications.

The aim of this study is to investigate the current causes of SD in a large population and to compare the aetiologies of SD between autopsied vs. resuscitated patients.

## Methods

We analysed and compared causes of SD in a large population associating both resuscitated patients and autopsied cases referred to our institution over the same period. Even if resuscitated patients and necropsies might come from different locations from our geographical area, we assume no difference in SD aetiologies according to the location.

Two distinct populations have been retrospectively studied.

1. The first population comprised all successive patients referred for resuscitated SD to our University Hospital along an eleven years period (2010–2021). In-hospital SD were also included, except iatrogenic or occurring during or shortly after cardiac catheterization or surgery. Deaths occurring over the final course of neoplasms, multi-morbid states, or end-stage respiratory/cardiac failure were also rejected. Full investigational data were available, leading to a causal diagnosis as precise and exact as possible based on currently available medical investigations. SD in coronary artery disease patients were classified as ‘ischaemic’ (in the setting of acute coronary event) or as ‘nonischaemic’ (in the presence of previous myocardial infarction without documentation of new acute coronary event).
2. The second population included all necropsies performed for unexpected SD over a period of nine years (2012–2021). These autopsies had been ordered by different courts from Midi-Pyrénées area and performed at our institution. Deaths from drowning/submersion, from intoxications or nonanalysable necropsies (advanced putrefaction), were not included, as well as traumatic, criminal, or suicidal deaths.

Gender and age were the only available clinical data in this population. Complete macroscopic examination of the whole body and heart had been done for each case. Heart dimensions and weight were noted. Pathological investigation was only performed in selected cases after request of legal departments (i.e. without medical indication).

Cardiac abnormalities at autopsy were classified as follows:

- Coronary artery disease: defined by the presence of recent coronary artery thrombosis (fresh and adherent thrombus) and/or significant epicardial coronary artery stenosis (>70% and/or stenting) (with/without area of myocardial necrosis, fibrosis, or purple areas) or by the presence of tissue necrosis at pathology. Acute myocardial infarction was diagnosed as a light area sometimes surrounded by a red border at macroscopic examination. Macroscopic investigation for acute coronary event was considered reliable, even without the presence of fresh thrombus, especially when performed >8 h after death, and also for remote myocardial infarction of sufficient size.
- Cardiomegaly was defined by heart weight > mean + SD of matched individuals^2^ or >4–5 g/kg body weight in patients <18 years old.^3^ Myocardial hypertrophy was defined by parietal diameter >15 mm. Dilated cardiomyopathy (DCM) was defined by cardiomegaly without myocardial hypertrophy.
- Arrhythmogenic right ventricular cardiomyopathy (ARVC) and myocarditis were diagnosed after pathological evaluation using accepted common criteria

In the absence of any evident cardiac or noncardiac cause after macroscopic and sometimes pathological evaluation, the death was considered unexplained.

According to the French ethic and regulatory law, retrospective studies based on usual care data do not need to be submitted to an ethical committee but have to be covered by reference methodology of the French National Commission for Informatics and Liberties (CNIL). Toulouse University Hospital signed a commitment of compliance to the reference methodology MR-004 of the CNIL. After evaluation, this study completing all the criteria is registered at the Toulouse University Hospital (number RnIPH 2024-62) and covered by the MR-004 (CNIL number: 2206723 v 0).

### Statistics

Continuous variables are reported as mean ± SD and compared with unpaired *t*-test. Categorical variables (%) were compared using χ^2^ test. Analysis and calculations were performed using StatView™ program (Abacus Concepts, Inc., Berkeley, CA 1992–1996, version 5.0). A *P* value < 0.05 was considered statistically significant for each analysis.

## Results

A total of 310 patients had been referred for resuscitated SD, and 2100 necropsies have been performed for SD at our institution for the considered time periods.

### Patients with resuscitated SD

#### SD related to coronary artery disease

Among the 310 resuscitated patients, 283 patients were ≥16 years old. From these, coronary artery disease was considered the cause of SD in 245 (87%), with a majority of males (*n* = 185, 76%), a mean age of 62 ± 14 years old, and a mean left ventricular ejection fraction (LVEF) of 40 ± 15%.

All had undergone coronary artery angiography at admission. An acute coronary artery thrombosis was present in 206/245 (84%) (LVEF 40 ± 15%) and all underwent short-term revascularization. An SD occurring on previous myocardial infarction because of ventricular fibrillation/tachycardia (VF/VT)—without additional acute coronary event—was diagnosed in 35/245 (14%) (LVEF 36 ± 12%). A diagnosis of variant angina with coronary vasospasm had been retained in the four remaining cases (1.5%) after positive ergonovine test and otherwise negative investigations, of whom two without coronary stenosis. No ANOCOR (anomalous aortic origin of the coronary arteries) or MINOCA (myocardial infarction with normal coronary arteries) were found in this population.

#### SD unrelated to coronary artery disease

Out of the 283 adults with resuscitated SD, 38 patients (13%) did not present with coronary artery disease (males 60%, 48 ± 18 years old). All had undergone coronary artery angiography at admission (only one patient with significant coronary artery stenosis unrelated to SD).

Diverse types of cardiomyopathies (CM) were found in 16 out of these 38 patients (42%) with a mean LVEF of 33 ± 11%: hypertrophic (*n* = 4) (HCM), dilated CM (*n* = 3), ARVC (*n* = 2), cardiac sarcoidosis (*n* = 2), wild-type TTR cardiac amyloidosis (*n* = 1), significant aortic stenosis (*n* = 1), significant aortic regurgitation (*n* = 1), tako-tsubo (*n* = 1), mitral valve dystrophy (*n* = 1). Neither tamponade or congenital heart disease was found in this population.

A purely arrhythmic cause of SD was diagnosed in 22 out of the 38 cases (58%) (primary electrical diseases) without any structural heart disease or extracardiac causes after complete investigations, with a majority of malignant ventricular arrhythmia of unknown cause in the presence of early repolarization (*n* = 7) or idiopathic VF (*n* = 6), while Brugada syndrome was diagnosed in 3, hypokalaemia-induced VF, and/or torsades-de-pointes in two and catecholaminergic polymorphic VT in one. In one patient, a toxic cause (ibrutinib) was suspected, while isolated infra-Hisian conduction disturbances had been considered as the cause of SD in two cases. No pre-excitation was found in this population.

### Necropsies for SD

From the 2100 necropsies for unexpected SD, 190 drownings (9%), 203 toxic causes (10%), and 399 nonevaluable necropsies (19%) (putrefaction) were excluded.

From the remaining 1308 necropsies, 50 subjects <16 years old were furthermore excluded, letting 1258 necropsies in adults for final analysis, of whom 17% (212 cases) had undergone pathological investigation.

#### SD related to coronary artery disease

From the 1258 autopsied SD, 602 (48%) have been considered to be related to coronary artery disease, with a mean age of 58 ± 14 years old and a majority of males (*n* = 484, 80%).

- A fresh coronary thrombus was found in 22% of the 602 coronary artery disease patients (*n* = 135) indicative of an acute ischaemic coronary event causing SD.
- A SD occurring on ischaemic heart disease without acute coronary event was considered in 452/602 cases (75%), without fresh coronary thrombus but with areas of remote myocardial infarction at macroscopic examination and coronary artery stenosis/stenting. Pathological evaluation had been performed in 68 cases (15%) with confirmation of the diagnosis in each case.
- In 12 cases, there were findings evocative of potential MINOCA.
- Two cases of myocardial infarctions by fat coronary thrombus/embolism.
- One isolated coronary artery dissection.
- One case of ANOCOR (right coronary artery arising from the left sinus of Valsalva with inter-aorto-pulmonary course).

Myocardial bridges were additionally reported in two patients (one with coronary stenosis, one with MINOCA).

#### SD not related to coronary artery disease

##### Cardiac causes

From the 1258 necropsies, various structural heart diseases were found in 266 (19.5%) (54 ± 17 years old, males 66%) (see details in *Table 1*).

**Table 1:** Detail of the 266 structural heart diseases found among the 1258 autopsies

Type of heart disease	nb (%)	Details
Hypertrophic CM	154 (58%)	Pathology in 70 (45%) 43 cases of adaptative LVH 17 cases of HCM 1 case of cardiac amyloidosis (wild-type TTR) 9 cases with minor/absent/nonspecific lesions
Dilated CM	55 (21%)	Pathology in 7 (13%)
Myocarditis/pericarditis/endocarditis	14 (5%)	No major pericardial effusion
ARVC	12 (4%)	All confirmed at pathological evaluation
Unexplained purple area	10 (3%)	
Valvular heart disease	9 (3%)	Aortic *n* = 3, mitral *n* = 2, both *n* = 2 (2 NA)
Left fibrotic/fatty cardiomyopathy	5 (2%)	
Unexplained cardiac perforation	3	
Cor pulmonare	2	COPD *n* = 1 and pulmonary infection *n* = 1
Congenital heart disease	1	Tetralogy of Fallot
Commotio cordis	1	

##### Extracardiac causes

Extracardiac causes for SD were found in 16% (*n* = 205):

- Pulmonary embolisms (4%, *n* = 50) (52% males, 48 +/−23 years old)
- Aortic dissections/aortic aneurysm ruptures (4%, *n* = 48) (58% males, 66 +/−16 years old)
- Strokes and brain/meningeal haemorrhages (8%, *n* = 105) (66% males, 60+/−11 years old)
- Asphyxia (*n* = 2)

##### Lack of cardiac or extracardiac explanations for SD after necropsy

Finally, sudden arrhythmic death syndrome was suspected in 185 subjects (15%) without apparent cardiac or noncardiac cause at necropsy, with normal pathological investigation in 42 (23%), with a mean age of 49+/−17 years old and a majority of males (*n* = 116, 63%). Concomitant extracardiac pathology may have been implicated in a very few (*n* = 3, hypo-parathyroidism, epilepsy, and anorexia nervosa), while a pre-excitation had been previously documented in one (considered responsible for SD in the absence of any other cause) and Marfan disease suspected in one (aortic media necrosis) but without dissection so that SD was considered unexplained.

##### Comparisons between resuscitated SD and necropsies

There were no significant differences in age and gender between resuscitated patients and autopsied cases.

**Figure 1:**
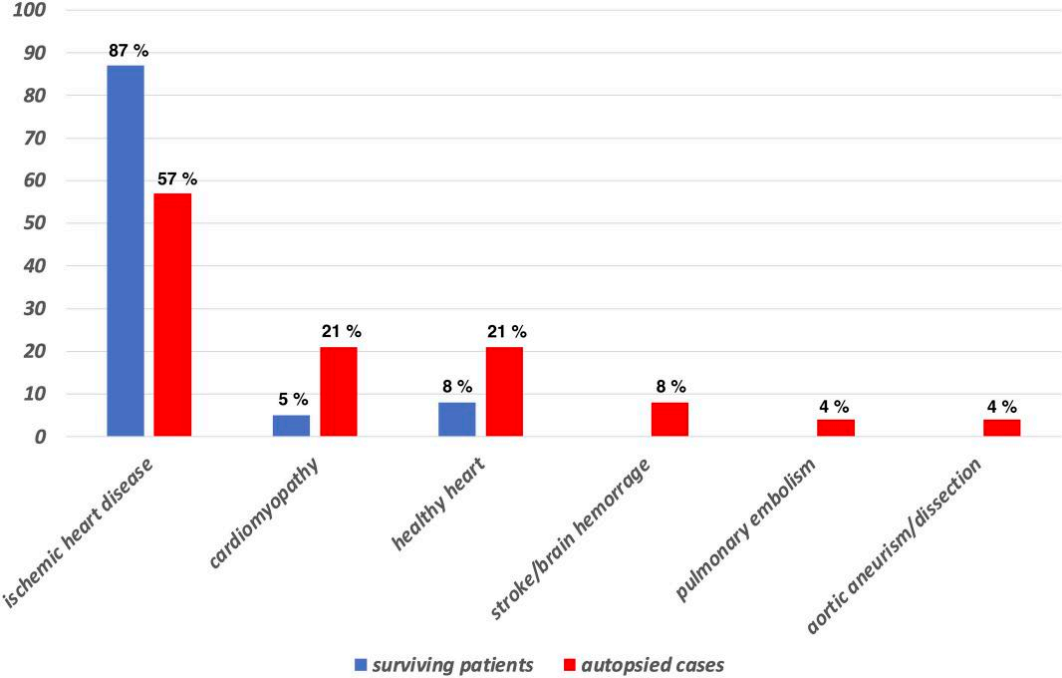
Causes for SD in surviving patients and autopsied cases.

Causes for SD were different between both populations. There was a complete lack of pulmonary embolisms, aortic dissections/aneurisms, and strokes/brain haemorrhages in resuscitated patients (*Figure 1*). We also found a higher predominance of coronary artery disease in resuscitated SD vs. a higher proportion of cardiomyopathy or unexplained SD in necropsies (*P* < 0.0001, with or without excluding pulmonary, aortic, and neurological causes).

**Figure 2:**
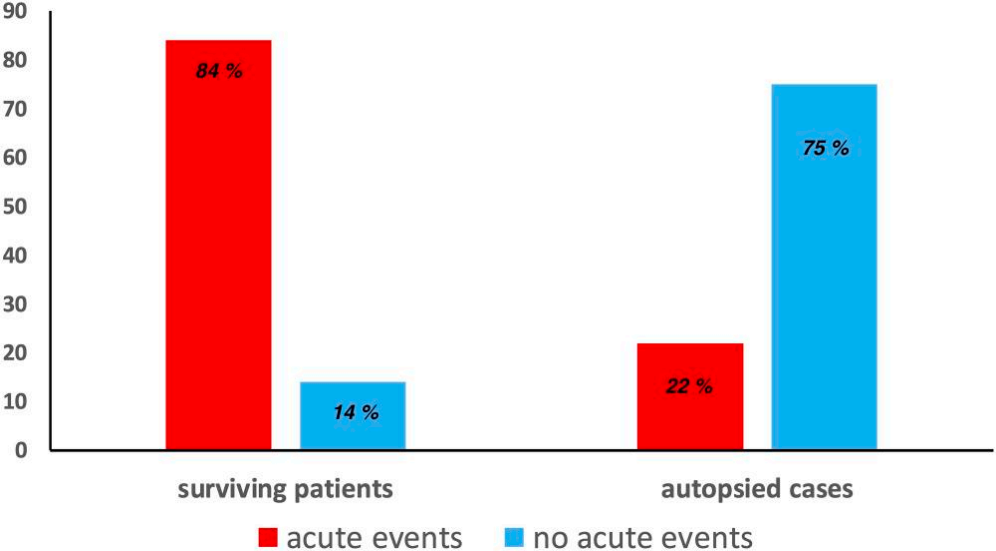
Causes for SD in coronary artery disease patients.

In the group of SD associated to coronary artery disease, after exclusion of coronary artery spasms, there was significantly more acute coronary events in resuscitated patients (85%) than in autopsied subjects (22%) (*P* < 0.0001) (*Figure 2*).

### SD in younger patients

In the 27 resuscitated young patients <16 years old, sudden infant death syndrome was noted in 5, diverse types of CM were found in 8, as well as respiratory causes (*n* = 8), channelopathy (*n* = 2, long QT and CPVT), neurological cases (*n* = 2, epilepsy and intracerebral bleeding), and 2 remained unexplained.

In the 50 autopsied patients <16 years old, 27 were sudden infant death syndrome, some CM were found in 16, and autopsy was negative in 6 (NA in one).

## Discussion

We report here the aetiologies of all successive surviving or autopsied patients experiencing SD and referred to our University Hospital over the last 10 years. With more than 300 resuscitated patients and more than 1200 autopsied cases, this is the largest series of investigated SD to date. As such, it could serve as a picture of the current causes of SD in unselected populations.

Several retrospective autopsy series from SD occurring in the general population have been published, including several hundreds of patients.^4,5^ There were also larger series but more ancient,^6^ whose results are possibly no more valid. Recently, a series of 899 autopsied cases was reported.^7^ The Fingesture study had collected data from 5869 consecutive autopsy in SD victims in Northern Finland, but no global results have been published to date.^8^

If aetiologies of SD have been previously reported in a wide number of autopsied case series, causes of SD in resuscitated patients do not seem to have been deeply investigated, or only in a limited number of selected cases^9^ and/or not separated from deceased patients.^10^ There is only one large prospective French registry including 1657 patients admitted after an aborted cardiac arrest.^11^ Thus, causes of SD in resuscitated patients remain grossly evaluated, and it is largely undetermined if causes are similar between deceased and surviving patients. Very recently, a vast Japanese registry comparing surviving and nonsurviving out-of-hospital cardiac arrests found more noncardiac causes and less acute coronary syndrome in nonsurvivors, but there was no autopsy in this study.^12^ A difference in aetiologies between deceased and resuscitated patients may have potential mechanistic and clinical implications. In this study, we investigate the current causes of SD in a large population, and we further compare the aetiologies of SD between autopsied vs. resuscitated patients.

Most of our results are comparable to what has been described before, although some differences should be noted.

### Coronary heart disease

Coronary artery disease has been considered responsible for the vast majority of resuscitated SD (87%). From these, an acute coronary event was found in most case (84%) and a malignant ventricular arrhythmia occurring on previous myocardial infarction (without acute coronary event) in a minority (14%), together with a few coronary artery spasms. Conversely, in autopsied patients, only half (48%) have been considered to be the result of coronary artery disease, with signs of acute coronary event in only 22% and an additional very small subset of other coronary artery abnormalities.

The clear prominence of coronary artery disease had been already repetitively mentioned in most series,^4,5,9,11,13,14^ reaching 64% in the recent large Brazilian series of autopsied SD,^7^ close to our findings.

Interestingly, coronary artery disease was present in most resuscitated SD but in only half of autopsied subjects. This significant lower proportion may be explained by the relevant part of noncardiac causes of SD in autopsied cases, by an imperfect sensitivity of necropsy for ischaemic heart diseases (see below) or by a possible better prognosis of SD in coronary artery disease patients, allowing more frequent successful reanimation. This latter hypothesis deserves further investigations.

Some other differences between resuscitated and autopsied cases are interesting to discuss. The major part of autopsied subjects with coronary artery disease revealed previous remote myocardial infarction without recent ischaemic event, while a majority of acute coronary events was observed among surviving patients with coronary artery disease. In other words, in coronary artery disease patients, SD was rather caused by scar-related VF/VT in deceased cases and by ischaemia-related events in surviving ones. Rather reverse higher proportions of acute coronary occlusions have been reported in some autopsy series.^15–18^ Historically, coronary thrombi were found at autopsy in 20–93% of ischaemic SD,^15^ thus with a high variance hindering to draw some conclusion. In resuscitated patients, coronary artery angiography revealed acute lesions in only 25–50% in previous series.^19–22^ However, less than half of SD due to coronary artery disease had evidence of acute event in a recent autopsy study,^8^ while 75–90% of coronary artery disease patients were acute coronary syndrome in large series of aborted cardiac arrests,^11,23^ similar to our findings. In the recent Japanese registry there was also less acute coronary syndrome in nonsurvivors, but there was no autopsy in this study.^12^

Although speculative, this difference could also be explained by a different prognosis of ischaemia-related vs. scar-related arrhythmias/events.

While we can assume the precise aetiologies/mechanisms in surviving patients with coronary artery-related SD, some limitations happen about the investigations of necropsied hearts. The presence of coronary fresh thrombus may not be always easily detected at visual inspection,^15,24,25^ while sensitivity of macroscopic investigation for demonstrating an acute coronary event was probably imperfect,^15^ for example, when SD occurred soon after coronary occlusion (< 8 h after) and/or in case of spontaneous thrombolysis and especially in the presence of previous myocardial infarction.

The remaining causes of coronary artery-related SD appear marginal. Vasospastic angina was judged responsible for 1.5% of surviving patients but was not evaluable in deceased patients. This uncommon cause of SD should not be neglected. Only one case of ANOCOR was noted in our population. This is in opposition to different studies mentioning rates of 2–4%,^26–28^ although some other works did also not find any case.^5,29^ Geographical/genetic disparities may possibly explain such differences.

Finally, MINOCA were suspected in a few deceased patients. Cases of histologically confirmed MI and normal coronary arteries have already been identified.^30^ However, such fibrosis areas may also be linked to some nonischaemic cardiomyopathies. Alternatively, minor culprit coronary artery lesions may have been overlooked at macroscopic investigation.

### Noncoronary artery diseases

In surviving patients, only 13% did not present with coronary artery disease, with diverse types of structural heart diseases in half and primary electrical diseases in the other half. In deceased subjects, noncoronary structural heart diseases were found in 18%, whereas SD was considered unexplained in 15% and extracardiac causes were found in the remaining 16%.

The proportion of HCM in our autopsied population is probably overestimated, even if previous series mentioned rates of 5–15%.^4,31–34^ Myocardial thickness may be also related to cadaveric rigidity.^35,36^ On the other hand, pathological and clinical correlations in HCM are imperfect^37,38^ and even unapparent ultra-structural changes without clear hypertrophy may cause SD. Moreover, adaptive LVH—whose relationship to SD remains to be proved—could not be differentiated from HCM in the absence of pathological investigation. In fact, true HCM was infrequent after pathological investigation in our series. Conversely, HCM were found in around 1% of resuscitated SD, which appears rather low regarding the relative high incidence of HCM.^39^ This low proportion should question about the true risk for SD in HCM, which could have been overestimated. The higher rate of HCM in necropsied vs. resuscitated SD may also raise possibility of a very bad outcome of SD when occurring in HCM.

Dilated CM was found in 1% of surviving patients and 4% of autopsied subjects. Higher rates of 12%^31^ and 5%^32^ have been mentioned in young subjects^5^ or even higher in developing countries,^34^ while inherited cardiomyopathies were found in 16% of young subjects with SD.^40^ Thus, dilated CM as a cause of SD may have been previously overlooked in view of our findings.

Valvular diseases were present in around 1% of cases. Different valvular diseases have already been found in 2–4% of SD,^4,6,34^ thus representing a rather minor cause for SD. Mitral valve dystrophy was found in only one surviving patient but may have been missed at autopsy. It has been however found in 12% of necropsies <65 years old.^6^

Proportion of ARVC was low in our population (0.5%) as in previous works (2.8%).^4^ A prevalence of 5–10% was noted in French and Italian series^6,29^; however, genetic background in some areas may explain this particularly high rate.^41,42^

Amyloidosis appears an exceptional cause for SD, despite its incidence in older populations, even if oldest patients could have been underrepresented here and if amyloidosis could have been missed at visual necropsy (not any case however after pathological evaluations). Not any case was however mentioned using pathology in a large series of elderly people with SD.^43^ Thus, amyloidosis should not be considered as a relevant cause of SD.

Cardiac infections (myocarditis/pericarditis/endocarditis) were observed in 1% of necropsies and in none of surviving patients. Even if rare, myocarditis is a well-known cause of SD, especially in athletes,^44^ which can be found in 1–3% of necropsies^4,6,24^ or even more in young subjects,^31,32,45^ but may finally represent a rather minor cause of SD in view of our data, even if the lack of systematic pathology may have overlooked minor forms of myocarditis. Tamponade as a cause of SD seems also exceptional. No cardiac tumour was found in our series, although present in 1% in other works.^4^

No obvious cause for SD was found in 8% of surviving patients (primary electrical diseases) and in 15% of necropsies, representing ‘sudden arrhythmic death syndrome’ or ‘sudden unexpected death,’ very likely caused by primary electrical diseases also. These values are in accordance with previous data,^11,26,31,46,47^ although most of all these series included only young subjects. Inherited cardiac conditions represented half of resuscitated patients of any age with unexplained SD.^9^

The role of isolated paroxysmal AV block and bradycardia in SD is probably marginal.^48^ Only two cases were diagnosed in resuscitated patients, while no evocative septal lesions were mentioned in autopsied hearts (although sensitivity is probably non optimal). His bundle lesions had been noted in up to 30% in large series, but often associated with other abnormalities.^6^

### Noncardiac causes

Interestingly, noncardiac causes of SD—which has been usually considered marginal—represented in fact a non-negligible part of necropsies, while being fully absent in resuscitated SD. Pulmonary embolisms, aortic dissections/ruptures of aneurisms, or strokes/brain/meningeal haemorrhages were noted in 4%, 4%, and 8%, respectively, of deceased patients, together with more exceptional asphyxial cases. This differentiates SD from SCD in this population. This may be explained by the fact that reanimation in presence of such causes is probably helpless once SD occurs, representing a final complication not prone to resuscitation. In the recent Japanese registry also there were more noncardiac causes in nonsurvivors, but there was no autopsy in this study.^12^ However, noncardiac causes were found in up to one-third of cases in a large series of aborted SD,^11^ which was never the case in our experience. One-third of SD has already also been found to be of noncardiac origin after necropsy,^26^ and pulmonary embolisms^4,6,32,46^ and aortic dissection/aneurisms^4,6,26,32^ or even asthma^32^ or pulmonary infection/chronic cor pulmonare^6,49^ were shown non-negligible causes of SD. Strokes^50^ and brain haemorrhages^51^ are a relevant cause for SD in Asia or in young subjects.^32^ Even if mentioning these noncardiac aetiologies is relatively uncommon in most series, extracardiac causes of SD should not be overlooked.

Epilepsy as a cause for SD (SUDEP) was evoked in only one autopsied case and never met in surviving patients; thus, this cause is probably marginal, although sometimes suspected in some young patients.^32^  ^,52^ SUDEP are usually associated with structural brain lesions,^53^ whereas deadly status epilepticus leads to brain ischaemic damage,^54^ both of which were not observed here. Central nervous system tumours were not found in our series but already reported in a very few autopsied SD.^55^

### Limitations

Genetic testing has not been done in most of our autopsied hearts. Pathogenic DNA variants have been found in 25% of unexplained SD,^40,56^ mainly in genes involved in channelopathy or CM. Molecular autopsy may have changed the ratio of unexplained SD, without modifying the predominant role of ischaemic heart disease however.

Around 80% of necropsy did not include pathological investigation, because this evaluation was only ordered by legal decisions. Thus, a possible part of noncoronary subjects may have revealed different causes would pathology have been done. Although the additional role of pathology in autopsy has been shown to be marginal,^57^ detailed pathological description of the 212 cases with microscopical investigations—especially in young subjects—and correlations with death reports are out of the scope of this study but deserve further work.

The number of necropsies done after drownings (overrepresented because a highly suspicious cause of death on a legal point of view) and of nonanalysable cases because of putrefaction—which leads to artefactual dilatation and tend to slim ventricular walls^35,36^—may also render some of our results debatable. However, there is no obvious reason to postulate that causes would differ between analysable and nonanalysable cases. For drownings, however, this rises the hypothesis of an undetermined part of long QT or CPVT.^1^

Finally, clinical history and circumstances of death for the cohort of autopsied cases were not available, hindering multivariate analysis. Retrieving such informations for this wide population would have been challenging and probably imprecise and largely incomplete.

## Conclusions

Diverse causes were depicted in this large current series or autopsied and resuscitated SD, and even if coronary artery disease remains the main aetiology for SD, it was significantly less common in autopsied subjects. Moreover, in coronary artery disease patients, SD was rather caused by scar-related malignant arrhythmias in deceased cases and by ischaemia-related events in surviving patients. Noncardiac causes as pulmonary embolisms, aortic, or neurological causes were only observed in autopsied cases.

## Lead author biography

Medical studies in Bordeaux University Internship in Cardiology at University Hospital Toulouse

Junior Physician in Cardiac Electrophysiology and Pacing during 2 years at University Hospital Toulouse and University Hospital Montpellier

Current fellowship in Cardiac Electrophysiology and Pacing at Clinique Pasteur, Toulouse

## References

[1] Zeppenfeld K, Tfelt-Hansen J, de Riva M, Winkel BG, Behr ER, Blom NA, Charron P, Corrado D, Dagres N, de Chillou C, Eckardt L, Friede T, Haugaa KH, Hocini M, Lambiase PD, Marijon E, Merino JL, Peichl P, Priori SG, Reichlin T, Schulz-Menger J, Sticherling C, Tzeis S, Verstrael A, Volterrani M, Cikes M, Kirchhof P, Abdelhamid M, Aboyans V, Arbelo E, Arribas F, Asteggiano R, Basso C, Bauer A, Bertaglia E, Biering-Sørensen T, Blomström-Lundqvist C, Borger MA, Čelutkienė J, Cosyns B, Falk V, Fauchier L, Gorenek B, Halvorsen S, Hatala R, Heidbuchel H, Kaab S, Konradi A, Koskinas KC, Kotecha D, Landmesser U, Lewis BS, Linhart A, Løchen ML, Lund LH, Metzner A, Mindham R, Nielsen JC, Norekvål TM, Patten M, Prescott E, Rakisheva A, Remme CA, Roca-Luque I, Sarkozy A, Scherr D, Sitges M, Touyz RM, Van Mieghem N, Velagic V, Viskin S, Volders PGA, Kichou B, Martirosyan M, Scherr D, Aliyev F, Willems R, Naser N, Shalganov T, Milicic D, Christophides T, Kautzner J, Hansen J, Allam L, Kampus P, Junttila J, Leclercq C, Etsadashvili K, Steven D, Gatzoulis K, Gellér L, Arnar DO, Galvin J, Haim M, Pappone C, Elezi S, Kerimkulova A, Kalejs O, Rabah A, Puodziukynas A, Dimmer C, Sammut MA, David L, Boskovic A, Moustaghfir A, Maass AH, Poposka L, Mjolstad OC, Mitkowski P, Parreira L, Cozma D, Golukhova E, Bini R, Stojkovic S, Hlivak P, Pernat A, Castellano NP, Platonov PG, Duru F, Saadi ARA, Ouali S, Demircan S, Sychov O, Slade A. 2022 ESC guidelines for the management of patients with ventricular arrhythmias and the prevention of sudden cardiac death. Eur Heart J 2022;43:3997–4126. 36017572 10.1093/eurheartj/ehac262

[2] De la Grandmaison GL, Clairand I, Durigon M. Organ weight in 684 adult autopsies: new tables for a Caucasoid population. Forensic Sci Int 2001;119:149–154. 11376980 10.1016/s0379-0738(00)00401-1

[3] Schoppen ZJ, Balmert LC, White S, Olson R, Arunkumar P, Dellefave-Castillo LM, Puckelwartz MJ, George AL, McNally EM, Webster G. Prevalence of abnormal heart weight after sudden death in people younger than 40 years of age. J Am Heart Assoc 2020;9:e015699. 32885733 10.1161/JAHA.120.015699 PMC7726998

[4] Mesrati MA, Belhadj M, Aissaoui A, HajSalem N, Oualha D, Boughattas M, Messaoudi I, Hammedi F, Zakhama A, Chadly A. Sudden cardiovascular death in adults: study of 361 autopsy cases. Ann Cardiol Angeiol 2017;66:7–14. 10.1016/j.ancard.2016.03.003 27109042

[5] Naneix AL, Périer MC, Beganton F, Jouven X, Lorin de la Grandmaison G. Sudden adult death: an autopsy series of 534 cases with gender and control comparison. J Forensic Leg Med 2015;32:10–15. 25882142 10.1016/j.jflm.2015.02.005

[6] Loire R, Tabib A. Mort subite cardiaque inattendue. Bilan de 1000 autopsies. Arch Mal Coeur Vaiss 1996;89:13–18. 8678733

[7] Braggion-Santos MF, Volpe GJ, Pazin-Filho A, Maciel BC, Marin-Neto JA, Schmidt A. Sudden cardiac death in Brazil: a community-based autopsy series (2006-2010). Arq Bras Cardiol 2015;104:120–127. 25424162 10.5935/abc.20140178 PMC4375655

[8] Holmström L, Juntunen S, Vähätalo J, Pakanen L, Kaikkonen K, Haukilahti A, Kenttä T, Tikkanen J, Viitasalo V, Perkiömäki J, Huikuri H, Myerburg RJ, Junttila J. Plaque histology and myocardial disease in sudden coronary death: the Fingesture study. Eur Heart J 2022;43:4923–4930. 36172703 10.1093/eurheartj/ehac533 PMC9748531

[9] Rucinski C, Winbo A, Marcondes L, Earle N, Stiles M, Stiles R, Hooks D, Neas K, Hayes I, Crawford J, Martin A, Skinner JR. A population-based registry of patients with inherited cardiac conditions and resuscitated cardiac arrest. J Am Coll Cardiol 2020;75:2698–2707. 32466885 10.1016/j.jacc.2020.04.004

[10] Byrne R, Constant O, Smyth Y, Callagy G, Nash P, Daly K, Crowley J. Multiple source surveillance incidence and aetiology of out-of-hospital sudden cardiac death in a rural population in the west of Ireland. Eur Heart J 2008;29:1418–1423. 18424446 10.1093/eurheartj/ehn155

[11] Geri G, Passouant O, Dumas F, Bougouin W, Champigneulle B, Arnaout M, Chelly J, Chiche J-D, Varenne O, Guillemet L, Pène F, Waldmann V, Mira J-P, Marijon E, Cariou A. Etiological diagnoses of out-of-hospital cardiac arrest survivors admitted to the intensive care unit: insights from a French registry. Resuscitation 2017;117:66–72. 28602955 10.1016/j.resuscitation.2017.06.006

[12] Yoshimura S, Tseng ZH, Yamada T, Nakao S, Yoshiya K, Park C, Nishimura T, Ishibe T, Yamakawa K, Kiguchi T, Kishimoto M, Ninomiya K, Ito Y, Sogabe T, Morooka T, Sakamoto H, Hironaka Y, Onoe A, Matsuyama T, Okada Y, Matsui S, Nishioka N, Kimata S, Kawai S, Makino Y, Zha L, Kiyohara K, Kitamura T, Iwami T. Underlying cause of out-of-hospital cardiac arrests in Japan in survivors versus nonsurvivors. J Am Heart Assoc 2025;14:e036968. 40240947 10.1161/JAHA.124.036968 PMC12184230

[13] Zheng ZJ, Croft JB, Giles WH, Mensah GA. Sudden cardiac death in the United States, 1989 to 1998. Circulation 2001;104:2158–2163. 11684624 10.1161/hc4301.098254

[14] Anderson RE, Hill RB, Broudy DW, Key CR, Pathak D. A population-based autopsy study of sudden, unexpected deaths from natural causes among persons 5 to 39 years old during a 12-year period. Hum Pathol 1994;25:1332–1340. 8001928 10.1016/0046-8177(94)90094-9

[15] Davies MJ, Bland JM, Hangartner JR, Angelini A, Thomas AC. Factors influencing the presence or absence of acute coronary artery thrombi in sudden ischaemic death. Eur Heart J 1989;10:203–208. 2707268 10.1093/oxfordjournals.eurheartj.a059467

[16] Davies MJ, Thomas A. Thrombosis and acute coronary-artery lesions in sudden cardiac ischemic death. N Engl J Med 1984;310:1137–1140. 6709008 10.1056/NEJM198405033101801

[17] Reichenbach DD, Moss NS, Meyer E. Pathology of the heart in sudden cardiac death. Am J Cardiol 1977;39:865–872. 871113 10.1016/s0002-9149(77)80041-6

[18] Virmani R, Burke AP, Farb A. Sudden cardiac death. Cardiovasc Pathol 2001;10:211–218. 11673058 10.1016/s1054-8807(01)00091-6

[19] Moutacalli Z, Georges JL, Ajlani B, Cherif G, El Beainy E, Gibault-Genty G, Blicq E, Charbonnel C, Convers-Domart R, Boutot F, Caussanel J-M, Lemaire B, Legriel S, Livarek B. Immediate coronary angiography in survivors of out-of-hospital cardiac arrest without obvious extracardiac cause: who benefits? Ann Cardiol Angeiol 2017;66:260–268. 10.1016/j.ancard.2017.09.008 29029774

[20] Spaulding CM, Joly LM, Rosenberg A, Monchi M, Weber SN, Dhainaut JF, Carli P. Immediate coronary angiography in survivors of out-of-hospital cardiac arrest. N Engl J Med 1997;336:1629–1633. 9171064 10.1056/NEJM199706053362302

[21] Martínez-Losas P, Salinas P, Ferrera C, Nogales-Romo MT, Noriega F, Del Trigo M, Núñez-Gil IJ, Nombela-Franco L, Gonzalo N, Jiménez-Quevedo P, Escaned J, Fernández-Ortiz A, Macaya C, Viana-Tejedor A. Coronary angiography findings in cardiac arrest patients with non-diagnostic post-resuscitation electrocardiogram: a comparison of shockable and non-shockable initial rhythms. World J Cardiol 2017;9:702–709. 28932359 10.4330/wjc.v9.i8.702 PMC5583543

[22] Lo YS, Cutler JE, Blake K, Wright AM, Kron J, Swerdlow CD. Angiographic coronary morphology in survivors of cardiac arrest. Am Heart J 1988;115:781–785. 3354406 10.1016/0002-8703(88)90879-4

[23] Waldmann V, Karam N, Rischard J, Bougouin W, Sharifzadehgan A, Dumas F, Narayanan K, Sideris G, Voicu S, Gandjbakhch E, Jost D, Lamhaut L, Ludes B, Plu I, Beganton F, Wahbi K, Varenne O, Megarbane B, Algalarrondo V, Extramiana F, Lellouche N, Celermajer DS, Spaulding C, Lafont A, Cariou A, Jouven X, Marijon E. Low rates of immediate coronary angiography among young adults resuscitated from sudden cardiac arrest. Resuscitation 2020;147:34–42. 31857140 10.1016/j.resuscitation.2019.12.005

[24] Myerburg RJ, Junttila MJ. Sudden cardiac death caused by coronary heart disease. Circulation 2012;125:1043–1052. 22371442 10.1161/CIRCULATIONAHA.111.023846

[25] Myers A, Dewar HA. Circumstances attending 100 sudden deaths from coronary artery disease with coroner’s necropsies. Br Heart J 1975;37:1133–1143. 1191428 10.1136/hrt.37.11.1133 PMC482930

[26] Winkel BG, Holst AG, Theilade J, Kristensen IB, Thomsen JL, Ottesen GL, Bundgaard H, Svendsen JH, Haunsø S, Tfelt-Hansen J. Nationwide study of sudden cardiac death in persons aged 1-35 years. Eur Heart J 2011;32:983–990. 21131293 10.1093/eurheartj/ehq428

[27] Eckart RE, Shry EA, Burke AP, McNear JA, Appel DA, Castillo-Rojas LM, Avedissian L, Pearse LA, Potter RN, Tremaine L, Gentlesk PJ, Huffer L, Reich SS, Stevenson WG. Sudden death in young adults: an autopsy-based series of a population undergoing active surveillance. J Am Coll Cardiol 2011;58:1254–1261. 21903060 10.1016/j.jacc.2011.01.049

[28] Maron BJ, Doerer JJ, Haas TS, Tierney DM, Mueller FO. Sudden deaths in young competitive athletes: analysis of 1866 deaths in the United States, 1980-2006. Circulation 2009;119:1085–1092. 19221222 10.1161/CIRCULATIONAHA.108.804617

[29] Corrado D, Basso C, Rizzoli G, Schiavon M, Thiene G. Does sports activity enhance the risk of sudden death in adolescents and young adults? J Am Coll Cardiol 2003;42:1959–1963. 14662259 10.1016/j.jacc.2003.03.002

[30] Silvanto A, de Noronha SV, Sheppard MN. Myocardial infarction with normal coronaries: an autopsy perspective. J Clin Pathol 2012;65:512–516. 22378828 10.1136/jclinpath-2011-200597

[31] Wisten A, Forsberg H, Krantz P, Messner T. Sudden cardiac death in 15-35-year olds in Sweden during 1992-99. J Intern Med 2002;252:529–536. 12472914 10.1046/j.1365-2796.2002.01038.x

[32] Puranik R, Chow CK, Duflou JA, Kilborn MJ, McGuire MA. Sudden death in the young. Heart Rhythm 2005;2:1277–1282. 16360077 10.1016/j.hrthm.2005.09.008

[33] Papadakis M, Sharma S, Cox S, Sheppard MN, Panoulas VF, Behr ER. The magnitude of sudden cardiac death in the young: a death certificate-based review in England and Wales. Europace 2009;11:1353–1358. 19700472 10.1093/europace/eup229

[34] Ndoye EHO, Diallo AM, Thiam I, Soumah MM, Dia SA, Ndiaye M. Sudden cardiac death in Dakar: epidemiological and anatomo-pathological characteristics. Forensic Med Anat Research 2019;7:51–61.

[35] Basso C, Michaud K, d’Amati G, Banner J, Lucena J, Cunningham K, Leone O, Vink A, van der Wal AC, Sheppard MN. Cardiac hypertrophy at autopsy. Virchows Arch Int J Pathol 2021;479:79–94. 10.1007/s00428-021-03038-0 PMC8298245 33740097

[36] Cunningham KS, Spears DA, Care M. Evaluation of cardiac hypertrophy in the setting of sudden cardiac death. Forensic Sci Res 2019;4:223–240. 31489388 10.1080/20961790.2019.1633761 PMC6713129

[37] Cui H, Schaff HV, Lentz Carvalho J, Nishimura RA, Geske JB, Dearani JA, Lahr BD, Lee AT, Bos JM, Ackerman MJ, Ommen SR, Maleszewski JJ. Myocardial histopathology in patients with obstructive hypertrophic cardiomyopathy. J Am Coll Cardiol 2021;77:2159–2170. 33926651 10.1016/j.jacc.2021.03.008

[38] Rowin EJ, Fifer MA. Evaluating histopathology to improve our understanding of hypertrophic cardiomyopathy. J Am Coll Cardiol 2021;77:2171–2173. 33926652 10.1016/j.jacc.2021.03.292

[39] Semsarian C, Ingles J, Maron MS, Maron BJ. New perspectives on the prevalence of hypertrophic cardiomyopathy. J Am Coll Cardiol 2015;65:1249–1254. 25814232 10.1016/j.jacc.2015.01.019

[40] Bagnall RD, Weintraub RG, Ingles J, Duflou J, Yeates L, Lam L, Davis AM, Thompson T, Connell V, Wallace J, Naylor C, Crawford J, Love DR, Hallam L, White J, Lawrence C, Lynch M, Morgan N, James P, du Sart D, Puranik R, Langlois N, Vohra J, Winship I, Atherton J, McGaughran J, Skinner JR, Semsarian C. A prospective study of sudden cardiac death among children and young adults. N Engl J Med 2016;374:2441–2452. 27332903 10.1056/NEJMoa1510687

[41] Corrado D, Basso C, Thiene G, McKenna WJ, Davies MJ, Fontaliran F, Nava A, Silvestri F, Blomstrom-Lundqvist C, Wlodarska EK, Fontaine G, Camerini F. Spectrum of clinicopathologic manifestations of arrhythmogenic right ventricular cardiomyopathy/dysplasia: a multicenter study. J Am Coll Cardiol 1997;30:1512–1520. 9362410 10.1016/s0735-1097(97)00332-x

[42] Nava A, Bauce B, Basso C, Muriago M, Rampazzo A, Villanova C, Daliento L, Buja G, Corrado D, Danieli GA, Thiene G. Clinical profile and long-term follow-up of 37 families with arrhythmogenic right ventricular cardiomyopathy. J Am Coll Cardiol 2000;36:2226–2233. 11127465 10.1016/s0735-1097(00)00997-9

[43] Puolitaival E, Vähätalo J, Holmström L, Haukilahti MAE, Pakanen L, Ukkola OH, Junttila MJ, Huikuri HV, Perkiömäki JS. Causes and characteristics of unexpected sudden cardiac death in octogenarians/nonagenarians. PLoS One 2023;18:e0284515. 37079646 10.1371/journal.pone.0284515 PMC10118134

[44] Harris KM, Mackey-Bojack S, Bennett M, Nwaudo D, Duncanson E, Maron BJ. Sudden unexpected death due to myocarditis in young people, including athletes. Am J Cardiol 2021;143:131–134. 33347841 10.1016/j.amjcard.2020.12.028

[45] Svane J, Lynge TH, Hansen CJ, Risgaard B, Winkel BG, Tfelt-Hansen J. Witnessed and unwitnessed sudden cardiac death: a nationwide study of persons aged 1-35 years. Europace 2021;23:898–906. 33595080 10.1093/europace/euab017

[46] Margey R, Roy A, Tobin S, O’Keane CJ, McGorrian C, Morris V, Jennings S, Galvin J. Sudden cardiac death in 14- to 35-year olds in Ireland from 2005 to 2007: a retrospective registry. Europace 2011;13:1411–1418. 21798877 10.1093/europace/eur161

[47] Vaartjes I, Hendrix A, Hertogh EM, Grobbee DE, Doevendans PA, Mosterd A, Bots ML. Sudden death in persons younger than 40 years of age: incidence and causes. Eur J Cardiovasc Prev Rehabil 2009;16:592–596. 19587604 10.1097/HJR.0b013e32832d555b

[48] Leclercq JF, Coumel P, Maison-Blanche P, Cauchemez B, Zimmermann M, Chouty F, Slama R. Mechanisms determining sudden death. A cooperative study of 69 cases recorded using the holter method. Arch Mal Coeur Vaiss 1986;79:1024–1033. 3096225

[49] Penttilä A . Sudden and unexpected natural deaths of adult males. An analysis of 799 forensic autopsies in 1976. Forensic Sci Int 1980;16:249–259. 7203323 10.1016/0379-0738(80)90210-8

[50] Tokashiki T, Muratani A, Kimura Y, Muratani H, Fukiyama K. Sudden death in the general population in okinawa: incidence and causes of death. Jpn Circ J 1999;63:37–42. 10084386 10.1253/jcj.63.37

[51] Murakoshi N, Aonuma K. Epidemiology of arrhythmias and sudden cardiac death in Asia. Circ J 2013;77:2419–2431. 24067274 10.1253/circj.cj-13-1129

[52] Siboni A, Simonsen J. Sudden unexpected natural death in young persons. Forensic Sci Int 1986;31:159–166. 3744209 10.1016/0379-0738(86)90183-0

[53] Thorn M . Neuropathologic findings in postmortem studies of sudden death in epilepsy. Epilepsia 1997;38:S32–S34. 10.1111/j.1528-1157.1997.tb06123.x 19909322

[54] Han BK, Towbin RB, De Courten-Myers G, McLaurin RL, Ball WS Jr. Reversal sign on CT: effect of anoxic/ischemic cerebral injury in children. AJNR Am J Neuroradiol 1989;10:1191–1198. 2512781 PMC8332434

[55] Eberhart CG, Morrison A, Gyure KA, Frazier J, Smialek JE, Troncoso JC. Decreasing incidence of sudden death due to undiagnosed primary central nervous system tumors. Arch Pathol Lab Med 2001;125:1024–1030. 11473451 10.5858/2001-125-1024-DIOSDD

[56] Priori SG, Blomström-Lundqvist C, Mazzanti A, Blom N, Borggrefe M, Camm J, Elliott PM, Fitzsimons D, Hatala R, Hindricks G, Kirchhof P, Kjeldsen K, Kuck K-H, Hernandez-Madrid A, Nikolaou N, Norekvål TM, Spaulding C, Van Veldhuisen DJ. 2015 ESC guidelines for the management of patients with ventricular arrhythmias and the prevention of sudden cardiac death: the task force for the management of patients with ventricular arrhythmias and the prevention of sudden cardiac death of the European Society of Cardiology (ESC). endorsed by: association for European paediatric and congenital cardiology (AEPC). Eur Heart J 2015;36:2793–2867. 26320108 10.1093/eurheartj/ehv316

[57] Chatelain D, Hebert A, Trouillet N, Charfi S, Stephens P, Manaouil C, Defouilloy C, Braconnier L, Jarde O, Sevestre H. Effectiveness of histopathologic examination in a series of 400 forensic autopsies. Ann Pathol 2012;32:4–13. 22325309 10.1016/j.annpat.2011.10.011

